# Case report: Safety and efficacy of synergistic treatment using selinexor and azacitidine in patients with atypical chronic myeloid leukemia with resistance to decitabine

**DOI:** 10.3389/fonc.2024.1353818

**Published:** 2024-02-07

**Authors:** Lu Liu, Xiaofeng Song, Wenhao Dong, Zhao Li, Dongmei Guo

**Affiliations:** ^1^ Department of Hematology, Qilu Hospital (Qingdao) of Shandong University, Qingdao, China; ^2^ Department of Hand and Foot Surgery, Qilu Hospital (Qingdao) of Shandong University, Qingdao, China

**Keywords:** atypical chronic myeloid leukemia, selinexor, azacitidine, case report, synergism

## Abstract

**Background:**

Atypical chronic myeloid leukemia (aCML) is a *BCR::ABL1* negative myelodysplastic/myeloproliferative neoplasm with poor overall survival. Some patients can be treated by allogeneic hematopoietic stem cell transplantation (allo-HSCT) from suitable donors. The effectiveness of decitabine or azacitidine (AZA) has recently been reported; however, their combined efficacy with selinexor has not yet been reported.

**Case description:**

In this study, we report the case of a patient with aCML who was successfully treated with selinexor combined with AZA. A 67-year-old man with a history of gastric mucosa-associated lymphoid tissue (MALT) lymphoma was admitted to the hospital with fatigue and emaciation. He was diagnosed with aCML and no longer responded to decitabine treatment after undergoing seven cycles. The patient was subsequently administered hydroxyurea (HU), selinexor, and AZA. After four courses of combination therapy, his blood cell counts improved; he no longer required transfusions and was able to discontinue HU. The patient continued receiving selinexor and AZA without severe complications. This case is the first to show that combinatorial selinexor and AZA therapy can effectively treat aCML.

**Conclusion:**

Our case sheds light on the importance of selinexor and AZA combined therapy in the exploration of new treatment strategies for aCML. Moreover, this treatment approach offers the possibility of bridging with allo-HSCT.

## 1 Introduction

Atypical chronic myeloid leukemia (aCML), *BCR::ABL1* negative, is a rare hematological malignancy categorized as myelodysplastic/myeloproliferative neoplasms (MDS/MPN) according to the World Health Organization (WHO) classification of myeloid neoplasms. aCML was initially described as an atypical form of *BCR::ABL1* positive chronic myeloid leukemia (CML), leading to confusion in terminology. Currently, aCML is defined entirely separately from CML (1, 2). Patients with aCML present with myeloproliferative features, including leukocytosis and splenomegaly, which can occur alongside anemia and thrombocytopenia. The white blood cell (WBC) count is at least 13 × 10^9^/L, with a minimum immature granulocyte proportion of 10% in the peripheral blood, including promyelocytes, myelocytes, and metamyelocytes. Severe dysgranulopoiesis is a characteristic of aCML (3). Although aCML has since been redesignated MDS/MPN with neutrophilia according to the WHO 2022 classification, the International Consensus Classification (ICC) of 2022 retains its original name. The criteria for aCML are summarized in **Table 1** (4, 5).

**Table 1:** Diagnostic criteria for aCML according to the WHO 2022 and ICC classifications.

Criteria	ICC 2022 classification	WHO 2022 classification
Nomenclature	Atypical chronic myeloid leukemia	Myelodysplastic/myeloproliferative neoplasm with neutrophilia
• White blood cell count	≥13 × 10^9^/L with immature^a^ myeloid cells constituting ≥10% of WBCs	≥13 × 10^9^/L with neutrophilia, with immature^a^ myeloid cells constituting ≥10% of WBCs
• Peripheral blood and bone marrow blasts	<20%	<20%
• Dysplasia	Dysgranulopoiesis; hyposegmented or hypersegmented neutrophils, with or without abnormal chromatin clumping	Circulating immature^a^ myeloid cells constituting ≥10% of WBCs, with neutrophilic dysplasia
• Monocytes	<10%	<10%
• Bone marrow cellularity and hematopoiesis	Hypercellular with granulocytic hyperplasia and granulocytic dysplasia, with or without involvement of other lineages	Hypercellular with granulocytic hyperplasia and granulocytic dysplasia, with or without involvement of other lineages
• Molecular exclusionary criteria	*BCR::ABL1* or tyrosine kinase fusions associated with myeloid/lymphoid neoplasms with eosinophilia. *JAK2*, *MPL*, and *CALR* mutations	*BCR::ABL1* or tyrosine kinase fusions associated with myeloid/lymphoid neoplasms with eosinophilia *JAK2*, *MPL*, and *CALR* mutations *CSF3R* mutations MDS/MPN-RS-T with *SF3B1* mutations
• Next-generation sequencing data	Desirable to document the presence of *ASXL1* and *SETBP1* mutations	Desirable to document the presence of *SETBP1* and/or *ETNK1* mutations

a Immature myeloid cells include promyelocytes, myelocytes, and metamyelocytes.

The most commonly mutated genes (>20%) in aCML are *SETBP1*, *ASXL1*, *N/K-RAS*, *SRSF2*, and *TET2*; meanwhile, *CBL*, *CSFR3*, *JAK2*, *EZH2*, and *ETNK1* are less frequently mutated (<10%) (6–14). Although they are not univocally disease-specific, mutations in *SETBP1* and *ETNK1* are most closely associated with aCML (11, 12, 15–17). Multiple studies have shown that mutations in *SETBP1* are associated with an adverse clinical presentation, i.e., a higher leukocyte count, lower hemoglobin (Hb) level, and thrombocytopenia, suggesting that it is not only relevant to leukemic oncogenesis but also provides important prognostic value (10). Several of these mutations affect the JAK-STAT, MAPK, and ROCK signaling pathways, targetable by inhibitors that are in clinical use and may contribute to the personalized treatment of patients with aCML who are unfit for allogeneic transplant.

Although no standard care has been defined for the treatment of aCML, most patients are treated with hydroxyurea (HU) or interferon (IFN)-α to control blood cell counts. Meanwhile, a recent study demonstrated the effectiveness of decitabine and azacitidine (AZA) (18–21). Herein, we report a novel case of a 67-year-old male patient with non-transplant aCML who was successfully treated with combined selinexor and AZA.

## 2 Case report

**Figure 1:**
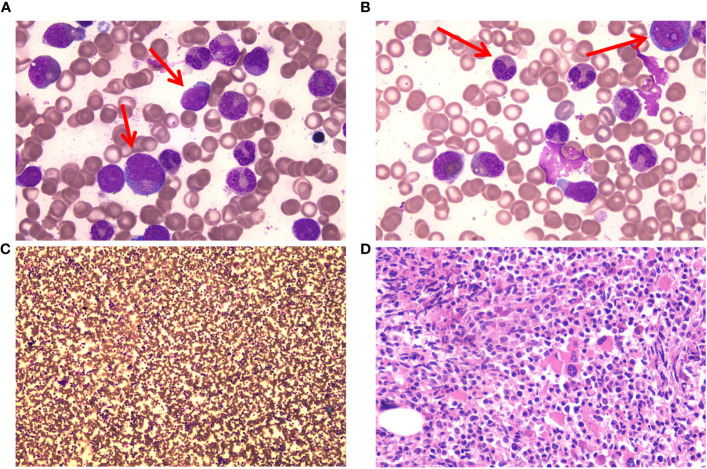
Bone marrow (BM) cells of the patient at the time of diagnosis. **(A)** Myeloblasts and promyelocytes in BM cell morphology. May–Gruenwald Giemsa stain, 100×. **(B)** Acquired Pelger–Huet anomaly and granulocytic dysplasia in BM cell morphology. May–Gruenwald Giemsa stain, 100×. **(C)** Hypercellular with granulocytic hyperplasia in BM cell morphology. May–Gruenwald Giemsa stain, 10×. **(D)** Hypercellular with granulocytic hyperplasia in BM biopsy. Hematoxylin-eosin stain. 40×.

A 67-year-old man with a history of MALT lymphoma presented to another hospital due to fatigue and emaciation. A blood test revealed a WBC count of 192 × 10^9^/L (blast cells 2.0%, neutrophilic myelocytes 21.0%, neutrophilic metamyelocytes 17.0%, monocytes 5%, eosinophils 0.1%, basophils 3.3%), a Hb concentration of 100 g/L, and a platelet (PLT) count of 95 × 10^9^/L. Additionally, L-lactate dehydrogenase and β2-microglobulin levels were high. Ultrasonography measured the splenomegaly (thickness: 63 mm; length: 183 mm). Morphological examination of the bone marrow revealed a granulocytic system/erythrocyte system (G/E) of 12.22/1. Granulocyte hyperplasia was extremely active, and obvious morphological abnormalities were observed (**Figure 1**). Immunophenotyping of the bone marrow showed 0.9% myeloblasts. A bone marrow biopsy revealed hypercellular with granulocytic hyperplasia and myelofibrosis (MF)-1. G-banding revealed a normal chromosomal karyotype, 46, XY [6], and fluorescence *in situ* hybridization showed that the patient did not have the *BCR::ABL1*, *PDGFRA*, *PDGFRB* fusion genes, or *FGFR1* reconstruction. Polymerase chain reaction results were *JAK2V617F-*, *JAK-EXON12/13-*, *MPL-*, *CALR-*, and *CSF3R-*negative. The patient was diagnosed with aCML and initiated on HU. Once the WBC count was controlled, the patient was administered decitabine.

Decitabine, the medicine approved by the China Food and Drug Administration, was intravenously administered (25 mg/m^2^/day) for 4 days at 4-week intervals. After seven cycles of treatment, morphological examination revealed hypercellular bone marrow (G/E = 3/1) and myeloblasts (12.8%). Immunophenotyping of the bone marrow revealed 12.3% myeloblasts. The response was categorized as progressive disease (PD). The patient discontinued therapy and was admitted to our hospital 2 months later.

We re-evaluated the bone marrow hyperplasia as active; 7% of myeloblasts and morphological abnormalities of granulocytes (pseudo-Pelger–Huet forms) were observed. Immunophenotyping of bone marrow revealed 14.6% myeloblasts. Bone marrow biopsies revealed hypercellular myeloblasts 10%, MF-3. G-banding revealed a normal 46, XY chromosome karyotype [20]. Laboratory experiments demonstrated a WBC count of 72.28 × 10^9^/L (blast cells 7.0%), a hemoglobin concentration of 43 g/L, and a PLT count of 35 × 10^9^/L. Next-generation sequencing identified the following pathogenic mutations: *SETBP1* c.2606G > A p.(S869N) VAF 50.50%; *ASXL1* c.1888_1910del p.(E635Rfs*15) VAF 41.70%; *NRAS* c.35G>A p.(G12D) VAF 25.70%; *CBL* c.111OA>T p.(L370F) VAF 36.70%; and *SRSF2* c.284C>A p.(P95H) VAF 52.30%. Splenomegaly exceeding the navel level was observed on abdominal ultrasound; the spleen was 82 mm thick and 284 mm in length. The Eastern Cooperative Oncology Group (ECOG) score was 3.

**Figure 2:**
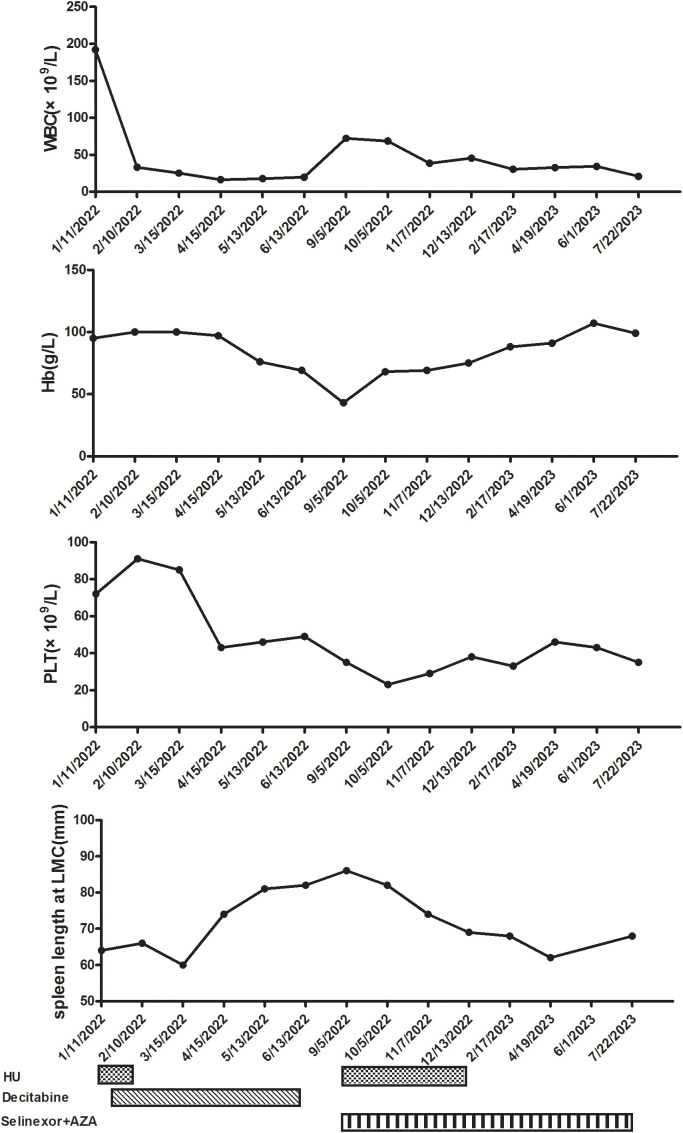
Clinical course after starting hydroxyurea (HU) and decitabine. WBC, white blood cell; Hb, hemoglobin; PLT, platelet; LMC, left midcostochondral line; AZA, azacitidine.

After communicating with the patient about his condition, he refused allogeneic hematopoietic stem cell transplantation (allo-HSCT) and signed an informed consent form for chemotherapy and over-the-counter administration of selinexor. We initiated treatment comprising HU, selinexor (40 mg/week), and AZA (7 days, 75 mg/m^2^). After one cycle, the patient did not experience any severe side effects, such as infection or fatigue, and night sweats resolved. The selinexor dose was subsequently increased to 60 mg/week. The patient experienced digestive tract side effects and lung infections; therefore, selinexor was scaled back to the previous dose. After the dosage reduction, the patient did not experience any further serious infections or gastrointestinal symptoms. After three cycles, we re-evaluated the bone marrow hyperplasia as active; the myeloblasts were at 3%, and granulocytic and erythroid dysplasia was detected. Laboratory experiments further revealed a WBC count of 45.31 × 10^9^/L (blast cell: 7.0%), a hemoglobin concentration of 75 g/L, and a PLT count of 46 × 10^9^/L. Fatigue symptoms were alleviated, and appetite and activity endurance improved. Due to the lack of validated response criteria for aCML, we defined the response as a partial marrow response based on the MDS/MPN International Working Group (MDS/MPN IWG) proposal (22). After four treatment courses, we stopped the HU and platelet transfusion. The patient prolonged the dosing interval of AZA on his own after recovery of physical status. To date, the patient has completed eight cycles of combination therapy with selinexor and AZA. We have re-evaluated the bone marrow hyperplasia as active, with 3% myeloblasts, 1% peripheral blood blasts, and abnormalities of granulocytes (pseudo-Pelger–Huet forms) and erythroid dysplasia observed morphologically. Immunophenotyping of the bone marrow revealed 3.16% myeloblasts. Bone marrow biopsies revealed no notable increases in hypercellularity and MF-3. Blood tests revealed a WBC count of 20.77 × 10^9^/L, a hemoglobin concentration of 99 g/L, and a PLT count of 35 × 10^9^/L (**Figure 2**). An abdominal ultrasound showed that the spleen was 62 mm thick and 198 mm in length, markedly smaller than at the beginning of treatment. The response was a partial marrow response, and there are clinical benefits for patients. The patient refused to undergo a review of the molecular indicators.

## 3 Discussion

aCML is an MDS/MPN overlap neoplasm characterized by dysplastic neutrophilia that occurs in the absence of monocytosis or eosinophilia (23). Most patients with aCML have an aggressive clinical course with a leukemic transformation in a significant subset (1, 2). Our patient presented with leukocytosis and neutrophilia, with immature myeloid cells (IMC), including promyelocytes, myelocytes, and metamyelocytes, comprising >10% of the WBCs. The characteristic feature that distinguishes aCML from other related MDS/MPN overlap neoplasms and MPN is the presence of marked dysgranulopoiesis, along with a high percentage of circulating IMC, without monocytosis (4, 5). Thus, defined and recurrent genetic abnormalities must be excluded (4, 5). Additionally, detection of *SETBP1* and/or *ETNK1* gene mutations is considered as desirable diagnostic criteria according to the 5th edition of the WHO classification (4). Meanwhile, the presence of *SETBP1* and *ASXL1* gene mutations in the absence of *CSF3R* supports aCML diagnosis according to the ICC (5). Given that the percentage of monocytes in our patient was <10% of the total leukocytes, the diagnostic criteria of CMML were not met (24). Patients with MDS/MPN-U may have spleen enlargement with thrombocythemia (>450 × 10^9^/L), and 20–30% are *JAK2V617F*-positive. Meanwhile, some patients present with aCML but without granulocyte dysplasia and with basophilic granulocytosis, megakaryocyte hyperplasia, and advanced myelofibrosis (24). Accordingly, our patient was diagnosed with aCML.

Gene mutations are present in most patients with aCML, with *ASXL1* mutations being the most common and linked to more aggressive disease. Meanwhile, *ETNK1* mutations are not specific to aCML. Ethanolamine kinase 1 (ETNK1) phosphorylates ethanolamine (Et) to phosphoethanolamine (P-Et), which participates in the main metabolic route by which cells synthesize critical cell membrane phospholipids (25). The most common *SETBP1* mutation is p. Gly870Ser, leading to increased SET binding protein 1 (SETBP1) and SET protein levels and decreased protein phosphatase 2A (PP2A) activity (12, 26). *SETBP1* also participates in gene transcription and histone modification, leading to gene regulation (12, 26). A recent series by Patnaik et al. demonstrated that increasing age, progressive anemia, and the presence of *TET2* mutations (but not *SETBP1*, *ASXL1*, and *ETNK1* mutations) were associated with worse outcomes. Based on these prognostic factors, they created a hazard ratio-weighted prognostic model and stratified patients into two risk categories: low (0–1 risk factor) and high (>2 risk factors). The median overall survival (OS) for the low-risk group was 18 months, whereas it was 7 months for the high-risk group (7). However, a study by Carreño-Tarragona et al. found that mutations in *CEBPA*, *EZH2*, *NRAS*, and *U2AF1* were associated with shorter survival, while *SRSF2* mutations were associated with better survival (27). Similarly, another study showed that *SRSF2* and *SETBP1* mutations were associated with improved OS, while *EZH2* and *RUNX1* mutations were associated with decreased OS (28). These findings do not fully agree with other studies, possibly due to the rarity and heterogeneity of these entities, which makes it challenging to assemble large cohorts. Therefore, their clinical impact should be validated in larger cohorts.

Due to the low incidence and absence of large clinical trials, management of aCML remains challenging. Most recommendations originate from small retrospective series and expert opinions. Indeed, several therapeutic strategies validated in other MDS/MPN cases have been applied to aCML. For example, the treatment of anemia and transfusion dependency is similar to that of other MDS/MPN entities treated with erythropoiesis-stimulating agent therapy or red blood cell transfusion. However, allo-HSCT may be the only curative option. The most common treatment is HU, which controls certain proliferative features, including leukocytosis and splenomegaly (29). Patients treated with IFN-α have also shown durable responses (29). Meanwhile, DNA methyltransferase inhibitors (DNMTis), AZA, and decitabine have been administered to patients with aCML, with limited success (7, 18). The largest amount of data has come from a study on 130 patients with *BCR::ABL1-*positive and -negative CML treated with decitabine (30). Among seven patients with aCML, four achieved a clinical response, including two complete hematological responses; however, the median survival was only 13 months, with an OS similar to that previously reported. Data on the use of AZA are very limited. Furthermore, *CSF3R* mutations are known to cause JAK pathway activation. Although uncommon, mutations in *CSF3R T618I* and *JAK2 V617F* have been identified in aCML, suggesting the potential effectiveness of JAK inhibitors. Both mutations activate the JAK–STAT pathway (31–33). As such, ruxolitinib, an oral JAK1/2 inhibitor, may be effective in treating this patient cohort. Meanwhile, fedratinib is a selective oral JAK2 and FLT3 inhibitor recently approved in the United States for the treatment of adult patients with intermediate-2 or high-risk MF (34). Fedratinib potently inhibits *FLT3* and *BRD4* and effectively suppresses c-MYC expression (35). It has been evaluated in a phase II study involving patients with MDS/MPN and chronic neutrophilic leukemia (CNL). The results demonstrated promising clinical efficacy in MDS/MPN and CNL patients with proliferative features (36). Additionally, a study by Carratt et al. revealed that *SETBP1* mutations promote self-renewal of *CSF3R*-mutant hematopoietic progenitors *in vitro* and prevent cells from undergoing terminal differentiation. *In vivo*, *SETBP1* mutations accelerate leukemia progression, leading to the rapid development of hepatosplenomegaly and granulocytosis. They also discovered that *SETBP1* upregulates *MYC* and *MYC* target genes. This increase in MYC expression can be reversed by lysine-specific demethylase 1 (LSD1) inhibitors. *SETBP1* mutations promote aggressive expansion of hematopoietic cells when expressed with mutant *CSF3R* through the upregulation of *MYC*-associated gene expression programs (37). For example, bomedemstat, an irreversible LSD1 inhibitor, may serve as a reference for new drug options for aCML. Trametinib is a specific inhibitor of mitogen-activated protein kinase kinase (MEK) 1–2, which downregulates extracellular signal-regulated kinase (ERK) in the mitogen-activated protein kinase (MAPK) pathway (38). Several reports have indicated that trametinib may be effective in patients with aCML with *RAS* mutations (39); however, these mutations exist in a few patients, and trametinib can be administered to a limited patient population. Allo-HSCT remains the only curative strategy for affected patients. Although some patients with a suitable donor can be treated with HSCT, due to increased age at presentation and comorbidities, most are not candidates for transplant. The European Society for Blood and Marrow Transplantation (EBMT) study confirmed that allo-HSCT represents a viable strategy to achieve a cure in a reasonable proportion of patients with aCML, particularly in young patients with low EBMT risk scores (2).

Selinexor inhibits nuclear protein export, and AZA is a demethylating drug. Both drugs regulate gene expression. Scattered data are available for unfit or very elderly patients for whom HMAs have failed. The response rate to decitabine after AZA is 30% (40–42). Due to the slightly different mechanisms of action between the two agents, switching may be partially justified. However, due to the low incidence, few pathophysiological studies have presented splenomegaly in aCML; such studies would prove highly informative. The expression of C-X-C motif chemokine ligand 12 (CXCL12), which is normally produced by osteoblasts and promotes the maintenance of HSCs, is increased in the spleen of patients with MF, leading to egress of hematopoietic elements from the bone marrow and localization in the spleen (43). Meanwhile, CXCR4—the canonical receptor for CXCL12—is downregulated in CD34^+^ cells from patients with MF secondary to hypermethylation, leading to decreased expression and likely egress from the HSC niche (44, 45). However, AZA-treated primary myelofibrosis (PMF) CD34^+^ cells exhibit a relatively complete reversal of CpG1 island hypermethylation and increased CXCR4 expression (45).

Additionally, selinexor has been evaluated in a phase 2 study of patients with resistance or intolerance to a JAK inhibitor at a dose of 60 to 80 mg weekly. A recently presented interim analysis of 12 patients revealed that the spleen volume response was 35% at 24 weeks, 30% in evaluable patients, and 40% at any time after 24 weeks. Symptom scores were not systematically collected. RBC transfusion independence was obtained in 2/5 dependent patients (46). Selinexor blocks XPO1, inducing nuclear localization of tumor suppressor proteins (including p53, p73, BRCA1, FOXO, and pRB), thereby leading to the selective induction of apoptosis and the inhibition of DNA damage repair proteins (47–49). In a phase II trial comprising 15 patients with relapsed/refractory AML treated with selinexor (50), a decline was observed in those with mutated *FLT3*, *SF3B1*, and *TP53* alleles, whereas clones with mutations in *CUX1*, *GATA2*, *TET2*, *BCOR*, *DNMT3A*, *RAD21*, *ASXL1*, *SRSF2*, *RUNX1*, *NPM1*, *PTPN11*, *ASXL2*, or *WT1* remained stable or increased under selinexor treatment. Therefore, the relationship between gene mutations in aCML and selinexor warrants further investigation.

An *in vitro* study reported that combined selinexor and AZA treatment synergistically induced cell cycle arrest, promoted apoptosis, and inhibited the proliferation of MDS cell lines. Furthermore, inhibition of XPO1 expression leads to increased p53 expression, which plays a key role in combination therapy-induced apoptosis (51). A previous study indicated that selinexor and AZA exert synergistic effects by suppressing XPO1/eIF4E/c-MYC signaling in patients with AML (52). Accordingly, based on these studies and our clinical findings for the case patient, we hypothesize that the spleen responded synergistically to AZA and selinexor. Collectively, these results provide pre-clinical evidence for further application of this novel combination.

In this case study, we have reported for the first time a patient with aCML who developed resistance to decitabine and exhibited a good response to combined selinexor and AZA treatment. The patient was able to continue using this therapy without severe complications. This novel treatment combination and mechanism merit further investigation in clinical trials. Moreover, this treatment approach offers the possibility of bridging with allo-HSCT. However, the follow-up time for this patient has been short. Hence, the patient’s prognosis, including whether he transforms to leukemia, and the efficacy duration, requires longer follow-up.

## 4 Patient perspective

The patient presented to the hospital due to fatigue and emaciation at the time of diagnosis. When the patient failed to respond to decitabine therapy, he refused to undergo allo-HSCT. After four courses of selinexor and AZA treatment, clinical benefits were noted for the patient. Fatigue symptoms were alleviated, and appetite and activity endurance improved. The patient prolonged the dosing interval of AZA on his own after physical status recovery. Due to the aggressive clinical course with leukemic transformation, we recommended regular treatment and close follow-up.

## Data availability statement

The datasets for this article are not publicly available due to concerns regarding patient anonymity. Requests to access the raw data should be directed to the corresponding authors.

## Ethics statement

The studies involving humans were approved by Medical Ethics Committee of Qilu Hospital (Qingdao) of Shandong University. The studies were conducted in accordance with the local legislation and institutional requirements. The participants provided their written informed consent to participate in this study. Written informed consent was obtained from the individual(s) for the publication of any potentially identifiable images or data included in this article.

## Author contributions

LL: Writing – original draft. XS: Data curation, Formal analysis, Writing – review & editing. WD: Data curation, Writing – review & editing. ZL: Supervision, Writing – review & editing. DG: Writing – review & editing.
